# 4-[2-(4-Fluoro­phen­yl)furan-3-yl]pyridine

**DOI:** 10.1107/S1600536809003651

**Published:** 2009-02-04

**Authors:** Bassam Abu Thaher, Pierre Koch, Dieter Schollmeyer, Stefan Laufer

**Affiliations:** a Faculty of Science, Chemistry Department, Islamic University of Gaza, Gaza Strip, Palestinian Territories; b Institute of Pharmacy, Department of Pharmaceutical and Medicinal Chemistry, Eberhard-Karls-University Tübingen, Auf der Morgenstelle 8, 72076 Tübingen, Germany; c Department of Organic Chemistry, Johannes Gutenberg-University Mainz, Duesbergweg 10-14, 55099 Mainz, Germany

## Abstract

In the crystal structure of the title compound, C_15_H_10_FNO, the furan ring makes dihedral angles of 40.04 (11) and 25.71 (11)° with the pyridine and 4-fluoro­phenyl rings, respectively. The pyridine ring makes a dihedral angle of 49.51 (10)° with the 4-fluoro­phenyl ring. Non-conventional C—H⋯F and C—H⋯N hydrogen bonds are effective in the stabilization of the crystal structure.

## Related literature

For the biological activities of related compounds, see: Wilkerson *et al.* (1985 ▶); Myers *et al.* (1985 ▶).
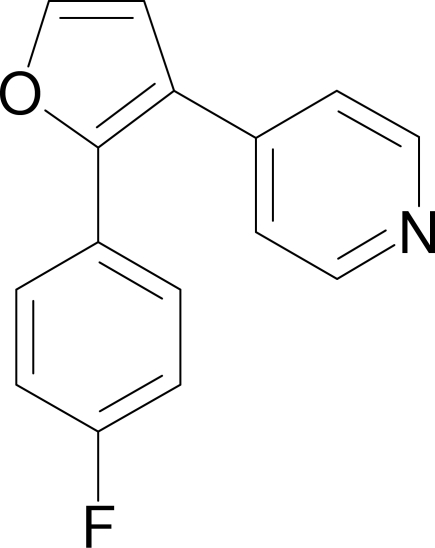


## Experimental

### 

#### Crystal data

C_15_H_10_FNO
*M* *_r_* = 239.24
Monoclinic, 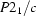

*a* = 13.343 (9) Å
*b* = 10.550 (3) Å
*c* = 8.178 (5) Å
β = 94.44 (3)°
*V* = 1147.7 (11) Å^3^
*Z* = 4
Cu *K*α radiation
μ = 0.81 mm^−1^
*T* = 193 (2) K
0.26 × 0.19 × 0.12 mm

#### Data collection

Enraf–Nonius CAD-4 diffractometer
Absorption correction: none
2172 measured reflections
2172 independent reflections
1806 reflections with *I* > 2σ(*I*)
3 standard reflectionsfrequency: 60 min intensity decay: 2%

#### Refinement

*R*[*F* ^2^ > 2σ(*F* ^2^)] = 0.052
*wR*(*F* ^2^) = 0.151
*S* = 1.07
2172 reflections
164 parameters
H-atom parameters constrained
Δρ_max_ = 0.23 e Å^−3^
Δρ_min_ = −0.31 e Å^−3^

### 

Data collection: *CAD-4 Software* (Enraf–Nonius, 1989 ▶); cell refinement: *CAD-4 Software*; data reduction: *CORINC* (Dräger & Gattow, 1971 ▶); program(s) used to solve structure: *SIR97* (Altomare *et al.*, 1999 ▶); program(s) used to refine structure: *SHELXL97* (Sheldrick, 2008 ▶); molecular graphics: *PLATON* (Spek, 2003 ▶); software used to prepare material for publication: *PLATON*.

## Supplementary Material

Crystal structure: contains datablocks I, global. DOI: 10.1107/S1600536809003651/bt2859sup1.cif

Structure factors: contains datablocks I. DOI: 10.1107/S1600536809003651/bt2859Isup2.hkl

Additional supplementary materials:  crystallographic information; 3D view; checkCIF report

## Figures and Tables

**Table 1:** Hydrogen-bond geometry (Å, °)

*D*—H⋯*A*	*D*—H	H⋯*A*	*D*⋯*A*	*D*—H⋯*A*
C5—H5⋯F1^i^	0.95	2.32	3.006 (3)	128
C8—H8⋯N15^ii^	0.95	2.60	3.483 (3)	155

Symmetry codes: (i) 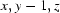; (ii) 
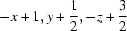
.

**Fig. 1.**
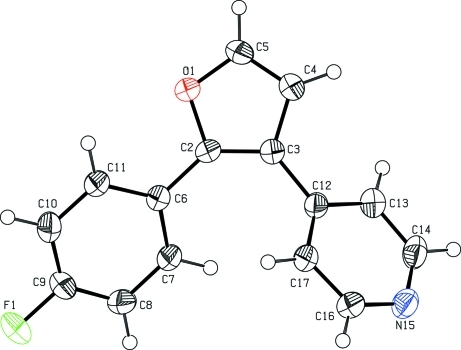
View of the title compound. Displacement ellipsoids are drawn at the 50% probability level. H atoms are depicted as circles of arbitrary size.

**Fig. 2.**
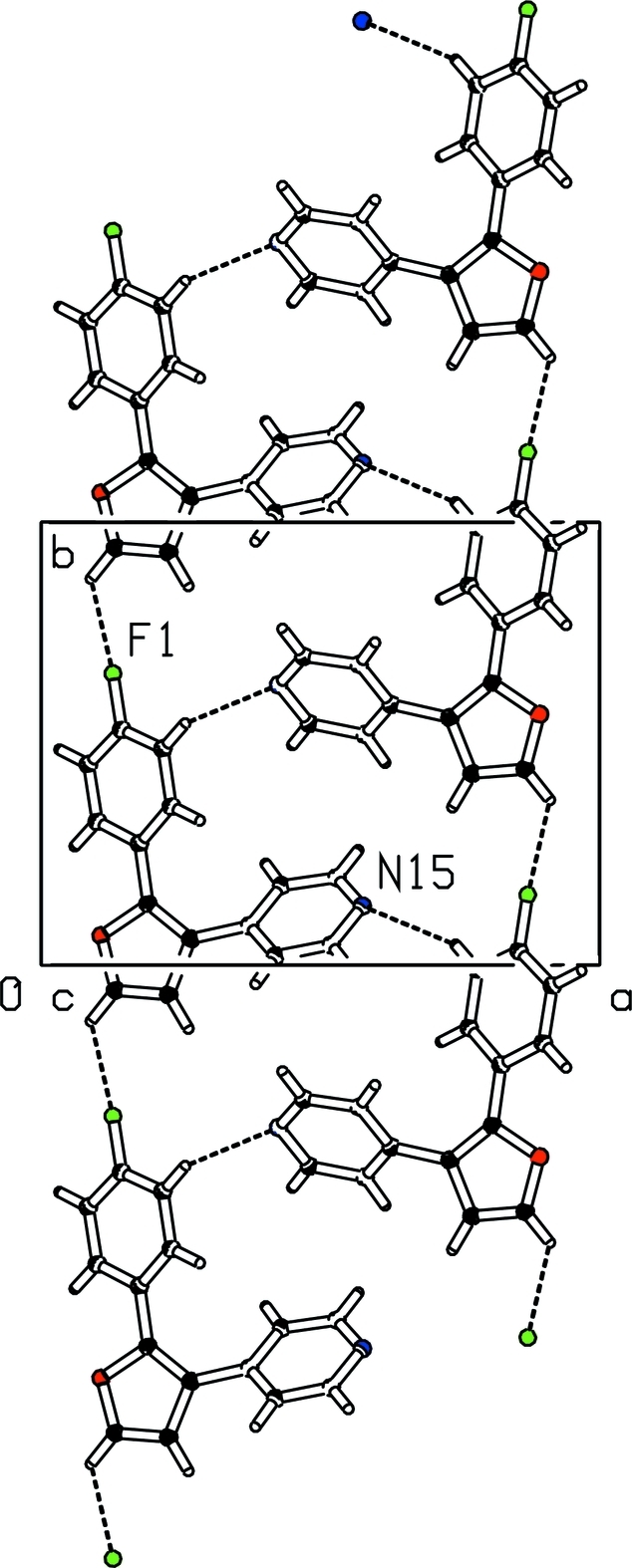
Partial crystal packing diagram of the title compound. The hydrogen bonds are shown with dashed lines. View along the c axis.

## supplementary crystallographic information

### Comment

Diarylfuran carbinols and methanamines (Wilkerson *et al.* 1985) and diaryl-thio-substituted furans (Myers *et al.* 1985) have been considered to be potential anti-inflammatory or analgetic agents.

The analysis of the crystal structure of the title compound is shown in Fig. 1. The furan ring makes dihedral angles of 40.04 (11)° and 25.71 (11)° to the pyridine ring and the 4-fluorophenyl ring, respectively. The pyridine ring makes a dihedral angle of 49.51 (10)° to the 4-fluorophenyl ring. Non-conventional C—H···*X* H-bonds seem to be effective in stabilization of the crystal structure. By intermolecular hydrogen bonds C5—H5···F1 (2.32 Å) and C8—H8···N15 (2.60 Å) a two-dimensional network parallel to the *ab* plane (Fig. 2) is formed.

### Experimental

4-(4-Fluorophenyl)-4-oxo-3-(pyridin-4-yl)butanal (2.0 g) was treated with glacial acetic acid (10 ml), conc. HCl (30 ml) and then heated to reflux temperature for 4 h. The reaction mixture was cooled to r.t. and put into ice. A solution of K_2_CO_3_ was added until it became basic. The aqueous phase was extracted four times with ethyl acetate and the combined organic layers were dried over Na_2_SO_4_ and filtered. The remaining solution was concentrated *in vacuo* and then purified by flash chromatography (SiO_2_, petroleum ether/ethylacetate 2:1 to 1:1) to give compound I (1.15 g) as a pale yellow solid. For X-ray suitable crystals of compound I were obtained by slow evaporation at 298 K of a solution of n-hexane–ethyl acetate–diethyl ether.

### Refinement

H atoms attached to carbons were placed at calculated positions with C—H = 0.95 Å. They were refined in the riding-model approximation with isotropic displacement parameters set to 1.2 times of the *U*_eq_ of the parent atom.

### Crystal data

**Table:**

C_15_H_10_FNO	*F*(000) = 496
*M**_r_* = 239.24	*D*_x_ = 1.385 Mg m^−^^3^
Monoclinic, *P*2_1_/*c*	Cu *K*α radiation, λ = 1.54178 Å
Hall symbol: -P 2ybc	Cell parameters from 25 reflections
*a* = 13.343 (9) Å	θ = 35–47°
*b* = 10.550 (3) Å	µ = 0.81 mm^−^^1^
*c* = 8.178 (5) Å	*T* = 193 K
β = 94.44 (3)°	Plate, colourless
*V* = 1147.7 (11) Å^3^	0.26 × 0.19 × 0.12 mm
*Z* = 4	

### Data collection

**Table:**

Enraf–Nonius CAD-4 diffractometer	*R*_int_ = 0.0000
Radiation source: rotating anode	θ_max_ = 70.1°, θ_min_ = 3.3°
graphite	*h* = 0→16
ω/2θ scans	*k* = 0→12
2172 measured reflections	*l* = −9→9
2172 independent reflections	3 standard reflections every 60 min
1806 reflections with *I* > 2σ(*I*)	intensity decay: 2%

### Refinement

**Table:**

Refinement on *F*^2^	Secondary atom site location: difference Fourier map
Least-squares matrix: full	Hydrogen site location: inferred from neighbouring sites
*R*[*F*^2^ > 2σ(*F*^2^)] = 0.052	H-atom parameters constrained
*wR*(*F*^2^) = 0.151	*w* = 1/[σ^2^(*F*_o_^2^) + (0.084*P*)^2^ + 0.3989*P*] where *P* = (*F*_o_^2^ + 2*F*_c_^2^)/3
*S* = 1.07	(Δ/σ)_max_ < 0.001
2172 reflections	Δρ_max_ = 0.23 e Å^−^^3^
164 parameters	Δρ_min_ = −0.31 e Å^−^^3^
0 restraints	Extinction correction: *SHELXL97* (Sheldrick, 2008), Fc^*^=kFc[1+0.001xFc^2^λ^3^/sin(2θ)]^-1/4^
Primary atom site location: structure-invariant direct methods	Extinction coefficient: 0.0023 (6)

### Special details

**Table:**

Geometry. All e.s.d.'s (except the e.s.d. in the dihedral angle between two l.s. planes) are estimated using the full covariance matrix. The cell e.s.d.'s are taken into account individually in the estimation of e.s.d.'s in distances, angles and torsion angles; correlations between e.s.d.'s in cell parameters are only used when they are defined by crystal symmetry. An approximate (isotropic) treatment of cell e.s.d.'s is used for estimating e.s.d.'s involving l.s. planes.
Refinement. Refinement of *F*^2^ against ALL reflections. The weighted *R*-factor *wR* and goodness of fit *S* are based on *F*^2^, conventional *R*-factors *R* are based on *F*, with *F* set to zero for negative *F*^2^. The threshold expression of *F*^2^ > σ(*F*^2^) is used only for calculating *R*-factors(gt) *etc*. and is not relevant to the choice of reflections for refinement. *R*-factors based on *F*^2^ are statistically about twice as large as those based on *F*, and *R*- factors based on ALL data will be even larger.

### Fractional atomic coordinates and isotropic or equivalent isotropic displacement parameters (Å^2^)

**Table:**

	*x*	*y*	*z*	*U*_iso_*/*U*_eq_	
F1	0.12844 (11)	0.65865 (12)	0.4490 (2)	0.0657 (5)	
O1	0.10747 (10)	0.06648 (13)	0.45018 (19)	0.0394 (4)	
C2	0.19141 (14)	0.13786 (18)	0.4958 (2)	0.0324 (4)	
C3	0.26801 (14)	0.05896 (18)	0.5490 (2)	0.0338 (5)	
C4	0.22829 (15)	−0.06710 (19)	0.5367 (3)	0.0403 (5)	
H4	0.2633	−0.1432	0.5656	0.048*	
C5	0.13306 (16)	−0.0573 (2)	0.4771 (3)	0.0438 (5)	
H5	0.0889	−0.1270	0.4561	0.053*	
C6	0.17617 (13)	0.27453 (18)	0.4833 (2)	0.0315 (4)	
C7	0.23331 (14)	0.35897 (19)	0.5837 (2)	0.0353 (5)	
H7	0.2837	0.3267	0.6610	0.042*	
C8	0.21809 (15)	0.4885 (2)	0.5730 (3)	0.0409 (5)	
H8	0.2577	0.5455	0.6408	0.049*	
C9	0.14411 (17)	0.53215 (19)	0.4616 (3)	0.0422 (5)	
C10	0.08431 (16)	0.4532 (2)	0.3621 (3)	0.0421 (5)	
H10	0.0329	0.4868	0.2876	0.051*	
C11	0.10057 (15)	0.32439 (19)	0.3728 (3)	0.0364 (5)	
H11	0.0601	0.2686	0.3046	0.044*	
C12	0.37309 (14)	0.08896 (18)	0.6061 (2)	0.0323 (4)	
C13	0.42007 (15)	0.0206 (2)	0.7362 (3)	0.0395 (5)	
H13	0.3841	−0.0426	0.7902	0.047*	
C14	0.51936 (16)	0.0457 (2)	0.7858 (3)	0.0425 (5)	
H14	0.5500	−0.0029	0.8739	0.051*	
N15	0.57492 (13)	0.13313 (18)	0.7186 (2)	0.0411 (5)	
C16	0.52945 (15)	0.1975 (2)	0.5926 (3)	0.0389 (5)	
H16	0.5676	0.2595	0.5405	0.047*	
C17	0.43063 (15)	0.17936 (19)	0.5335 (3)	0.0356 (5)	
H17	0.4024	0.2284	0.4439	0.043*	

### Atomic displacement parameters (Å^2^)

**Table:**

	*U*^11^	*U*^22^	*U*^33^	*U*^12^	*U*^13^	*U*^23^
F1	0.0692 (10)	0.0266 (7)	0.0986 (13)	0.0065 (6)	−0.0102 (8)	0.0003 (7)
O1	0.0293 (7)	0.0296 (7)	0.0575 (9)	−0.0026 (5)	−0.0079 (6)	−0.0016 (6)
C2	0.0274 (9)	0.0303 (10)	0.0383 (11)	−0.0035 (7)	−0.0038 (7)	−0.0019 (8)
C3	0.0310 (10)	0.0287 (10)	0.0406 (11)	0.0007 (8)	−0.0030 (8)	−0.0003 (8)
C4	0.0357 (11)	0.0289 (10)	0.0554 (13)	0.0023 (8)	−0.0017 (9)	0.0011 (9)
C5	0.0375 (11)	0.0253 (10)	0.0679 (15)	−0.0024 (8)	−0.0004 (10)	−0.0030 (9)
C6	0.0277 (9)	0.0306 (10)	0.0355 (10)	0.0014 (7)	−0.0019 (7)	−0.0001 (8)
C7	0.0308 (10)	0.0344 (11)	0.0392 (11)	0.0012 (8)	−0.0072 (8)	−0.0023 (8)
C8	0.0348 (11)	0.0337 (11)	0.0532 (13)	−0.0034 (8)	−0.0034 (9)	−0.0081 (9)
C9	0.0418 (12)	0.0264 (10)	0.0581 (14)	0.0035 (8)	0.0018 (9)	0.0009 (9)
C10	0.0396 (11)	0.0383 (12)	0.0467 (12)	0.0074 (9)	−0.0085 (9)	0.0033 (9)
C11	0.0327 (10)	0.0341 (10)	0.0410 (11)	0.0004 (8)	−0.0073 (8)	−0.0023 (8)
C12	0.0296 (10)	0.0293 (10)	0.0371 (10)	0.0037 (7)	−0.0035 (7)	−0.0040 (8)
C13	0.0373 (11)	0.0366 (11)	0.0438 (12)	0.0038 (8)	−0.0023 (9)	0.0042 (9)
C14	0.0389 (12)	0.0474 (12)	0.0396 (11)	0.0108 (9)	−0.0073 (9)	0.0016 (9)
N15	0.0345 (9)	0.0451 (11)	0.0420 (10)	0.0036 (7)	−0.0070 (7)	−0.0049 (8)
C16	0.0335 (10)	0.0380 (11)	0.0444 (11)	−0.0027 (8)	−0.0023 (8)	−0.0028 (9)
C17	0.0334 (10)	0.0341 (10)	0.0379 (10)	0.0011 (8)	−0.0071 (8)	0.0017 (8)

### Geometric parameters (Å, °)

**Table:**

F1—C9	1.354 (2)	C8—H8	0.9500
O1—C5	1.363 (2)	C9—C10	1.375 (3)
O1—C2	1.377 (2)	C10—C11	1.378 (3)
C2—C3	1.363 (3)	C10—H10	0.9500
C2—C6	1.459 (3)	C11—H11	0.9500
C3—C4	1.432 (3)	C12—C17	1.387 (3)
C3—C12	1.478 (3)	C12—C13	1.393 (3)
C4—C5	1.329 (3)	C13—C14	1.381 (3)
C4—H4	0.9500	C13—H13	0.9500
C5—H5	0.9500	C14—N15	1.329 (3)
C6—C7	1.397 (3)	C14—H14	0.9500
C6—C11	1.403 (3)	N15—C16	1.339 (3)
C7—C8	1.384 (3)	C16—C17	1.382 (3)
C7—H7	0.9500	C16—H16	0.9500
C8—C9	1.370 (3)	C17—H17	0.9500
			
C5—O1—C2	106.97 (16)	C8—C9—C10	123.0 (2)
C3—C2—O1	109.06 (17)	C9—C10—C11	118.58 (19)
C3—C2—C6	136.31 (18)	C9—C10—H10	120.7
O1—C2—C6	114.55 (16)	C11—C10—H10	120.7
C2—C3—C4	106.28 (17)	C10—C11—C6	120.82 (19)
C2—C3—C12	129.79 (18)	C10—C11—H11	119.6
C4—C3—C12	123.92 (18)	C6—C11—H11	119.6
C5—C4—C3	106.96 (18)	C17—C12—C13	116.85 (18)
C5—C4—H4	126.5	C17—C12—C3	123.72 (18)
C3—C4—H4	126.5	C13—C12—C3	119.38 (18)
C4—C5—O1	110.72 (18)	C14—C13—C12	119.4 (2)
C4—C5—H5	124.6	C14—C13—H13	120.3
O1—C5—H5	124.6	C12—C13—H13	120.3
C7—C6—C11	118.14 (18)	N15—C14—C13	124.34 (19)
C7—C6—C2	121.48 (17)	N15—C14—H14	117.8
C11—C6—C2	120.34 (17)	C13—C14—H14	117.8
C8—C7—C6	121.47 (18)	C14—N15—C16	115.86 (18)
C8—C7—H7	119.3	N15—C16—C17	124.2 (2)
C6—C7—H7	119.3	N15—C16—H16	117.9
C9—C8—C7	117.94 (19)	C17—C16—H16	117.9
C9—C8—H8	121.0	C16—C17—C12	119.35 (19)
C7—C8—H8	121.0	C16—C17—H17	120.3
F1—C9—C8	118.7 (2)	C12—C17—H17	120.3
F1—C9—C10	118.2 (2)		
			
C5—O1—C2—C3	0.5 (2)	C7—C8—C9—C10	0.7 (4)
C5—O1—C2—C6	−176.93 (18)	F1—C9—C10—C11	179.2 (2)
O1—C2—C3—C4	−0.6 (2)	C8—C9—C10—C11	−1.2 (4)
C6—C2—C3—C4	176.0 (2)	C9—C10—C11—C6	0.3 (3)
O1—C2—C3—C12	178.1 (2)	C7—C6—C11—C10	1.0 (3)
C6—C2—C3—C12	−5.3 (4)	C2—C6—C11—C10	178.8 (2)
C2—C3—C4—C5	0.5 (3)	C2—C3—C12—C17	−40.6 (3)
C12—C3—C4—C5	−178.3 (2)	C4—C3—C12—C17	138.0 (2)
C3—C4—C5—O1	−0.3 (3)	C2—C3—C12—C13	142.0 (2)
C2—O1—C5—C4	−0.1 (3)	C4—C3—C12—C13	−39.4 (3)
C3—C2—C6—C7	−24.2 (4)	C17—C12—C13—C14	0.1 (3)
O1—C2—C6—C7	152.23 (18)	C3—C12—C13—C14	177.75 (19)
C3—C2—C6—C11	158.1 (2)	C12—C13—C14—N15	0.8 (3)
O1—C2—C6—C11	−25.5 (3)	C13—C14—N15—C16	−1.4 (3)
C11—C6—C7—C8	−1.5 (3)	C14—N15—C16—C17	1.2 (3)
C2—C6—C7—C8	−179.28 (19)	N15—C16—C17—C12	−0.4 (3)
C6—C7—C8—C9	0.7 (3)	C13—C12—C17—C16	−0.3 (3)
C7—C8—C9—F1	−179.7 (2)	C3—C12—C17—C16	−177.80 (19)

### Hydrogen-bond geometry (Å, °)

**Table:**

*D*—H···*A*	*D*—H	H···*A*	*D*···*A*	*D*—H···*A*
C5—H5···F1^i^	0.95	2.32	3.006 (3)	128
C8—H8···N15^ii^	0.95	2.60	3.483 (3)	155

Symmetry codes: (i) *x*, *y*−1, *z*; (ii) −*x*+1, *y*+1/2, −*z*+3/2.
